# Cyclic tensile strain facilitates proliferation and migration of human aortic smooth muscle cells and reduces their apoptosis via miRNA-187-3p

**DOI:** 10.1080/21655979.2021.2009321

**Published:** 2021-12-11

**Authors:** Di Yang, Guang-Yuan Wei, Min Li, Ming-Sheng Peng, Yuan Sun, Yan-Liang Zhang, Chuang Lu, Kai-Xiong Qing, Hong-Bo Cai

**Affiliations:** a Department of Ophthalmology, The First Affiliated Hospital of Kunming Medical University, Kunming, Yunnan, China; b Department of Vascular Surgery, The First Affiliated Hospital of Kunming Medical University, Kunming, Yunnan, China

**Keywords:** Cardiovascular disorders, biomechanics, bioinformatics, cyclic tensile strain, human aortic smooth muscle cell, miRNAs

## Abstract

The cardiovascular is a system that contains extremely complex mechanical factors, in which the circulatory flow of blood has rich mechanical laws. Many studies have revealed that mechanical factors play a very important role in the process of revascularization. Hence, it is essential to investigate the mechanical factors in the process of revascularization in depth. A cyclic tensile strain (CTS) was applied to human aortic smooth muscle cells (HASMCs) at a frequency of 1 Hz and amplitudes of 5%, 10% and 15%, respectively. SmallRNA-seq was used to identify differentially expressed miRNAs (DE-miRNAs) responding to CTS in HASMCs. Starbase database predicted the target genes of DE-miRNAs. Metascape was applied for GO and KEGG pathway enrichment analysis and protein–protein interaction network construction. The proliferation and migration of CTS-treated HASMCs were significantly enhanced, and apoptosis were significantly reduced compared to the control group. SmallRNA-seq results demonstrated that 55, 16 and 16 DE-miRNAs were present in 5%, 10% and 15% CTS-treated HASMCs, respectively. Compared to controls, with miR-26a-2-3p and miR-187-3p being the intersection of these DE-miRNAs. Starbase database identified 189 common target genes for miR-26a-2-3p and miR-187-3p. Common target genes are mainly enriched in the basolateral plasma membrane and endocytosis. Further, *in vitro* experiments exhibited that CTS upregulated miR-187-3p expression, and miR-187-3p enhanced the proliferation and migration of HASMCs and reduced their apoptosis. It is suggested that miR-187-3p may be an important target for CTS participate in the process of cardiovascular disease.

**Figure:**
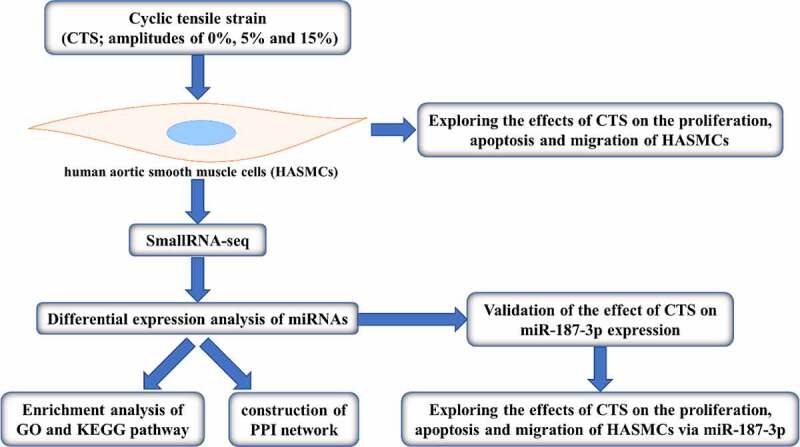

## Introduction

Cardiovascular disease is one of the diseases that seriously endanger human life and health, including hypertension, atherosclerosis, stroke, myocardial infarction, and aortic dissection, etc [1]. Cardiovascular disease as the most widespread of the non-communicable diseases, with an estimated 17.8 million deaths from the disease between 1980 and 2017 [2]. Notably, China has the largest proportion of countries at high risk for fatal cardiovascular disease, with approximately 33% of men and 28% of women having a 10% or greater risk of developing fatal cardiovascular disease within 10 years [3]. Abnormal changes in migration, proliferation, apoptosis and hypertrophy of vessel smooth muscle cells (VSMCs) are common to the pathogenesis and pathology of cardiovascular diseases, and are accompanied by changes in cell phenotype, morphology and structure, which subsequently lead to revascularization [4,5]. Hence, an advanced study of the phenotypic regulation of VSMCs is essential for the clarification of the development and treatment of cardiovascular diseases.

Biomechanical factors have been found to have a direct and significant impact on the onset and development of revascularization [6–8]. The vessel wall is subjected to mechanical forces in at least three different directions under *in vivo* conditions: cyclic tensile strain (CTS) caused by the natural pulsation of the blood, shear stress parallel to the direction of blood flow and pressure caused by the hydrostatic pressure of the blood flow [9,10]. Many studies have confirmed that CTS can cause changes in the structure of blood vessel walls, and can regulate the proliferation, apoptosis and migration of vascular cells, thereby having an important impact on the synthesis, degradation and reorganization of extracellular matrix [11–13]. VSMCs in the vascular media play a very important role in carrying CTS, and VSMCs can change cell functions in response to CTS stimulation [14,15]. When the vessel wall is subjected to high CTS caused by abnormally high blood pressure, VSMCs become hypertrophic and convert to a synthetic phenotype and undergo abnormal proliferation, resulting in thickening of the vessel wall, upregulation of elastin and collagen content, interstitial fibrosis, and increased vessel stiffness [16,17]. Therefore, an in-depth study of the mechanobiology of VSMCs, and finding the effect target of mechanical stress have very important theoretical and clinical significance for revealing the occurrence and development mechanism of vascular remodeling, as well as the prevention, and treatment of cardiovascular diseases.

MicroRNAs (miRNAs) are a class of non-coding single-stranded RNA that are approximately 20–24 nucleotides long and can be involved in post-transcriptional level regulation of gene expression [18]. Mature miRNAs are able to degrade or inhibit the translation of target mRNAs by specifically binding to the 3ʹUTR of homologous mRNAs [19,20]. miRNAs are involved in the regulation of a variety of biological functions, including cell differentiation, proliferation, metabolism and angiogenesis, among other processes [21,22]. Increasing evidence suggests that miRNAs play an important role in regulating VSMC biological behavior. For example, miR-145 was shown to be a major determinant of VSMC differentiation and phenotypic transition and was downregulated in both atherosclerosis and arterial injury models [23,24]. miR-125b inhibits VSMC calcification by targeting Ets1 [25]. Previous studies have also shown that CTS is involved in a variety of progressive processes by regulating the expression of cellular miRNAs. For example, CTS promotes differentiation of bone marrow mesenchymal stem cells into cardiomyocyte-like cells via miRNA-27a [26]. CTS promotes extracellular matrix synthesis in skull-base chondrocytes through upregulation of miR-140-5p [27]. However, the role of miRNAs in the regulation of the biological behavior of VSMCs by CTS remains to be investigated.

In this study, we elucidated for the first time the differentially expressed miRNAs in human aortic smooth muscle cells (HASMCs) in response to CTS by SmallRNA-seq. In addition, we selected miR-187-3p for *in vitro* validation of the response to CTS and the effect on the phenotype of HASMCs. In conclusion, the findings of this study will provide effector targets and therapeutic ideas for the application of CTS in cardiovascular diseases.

## Materials and methods

### Cell treatment

HASMCs were provided by the American Type Culture Collection (catalog number: PCS-100-012; Organism: *Homo sapiens*; Age: adult; Gender: Lot-specific; Disease: Normal). HASMCs were cultured in Dulbecco’s modified Eagle medium (DMEM; Sigma-Aldrich, USA) containing 10% fetal bovine serum (FBS; Gibco, USA), 100 U/mL penicillin, and 100 μg/mL streptomycin.

### Cell processing

As mentioned earlier [26,28,29], the FX-5000T Strain Unit Cellular Tension Strain Loading System was used to apply CTS to HASMCs. In brief, 4th-7th generation HASMCs were selected for trypsin digestion. HASMCs were inoculated into sterile Flexercell 6-well plates with a content of 2 × 10^5^/well. Then, Flexercell 6-well plates mounted in the FX-5000 TStrainUnit Cell Tension Loading System (Flexercell, USA). CTS was applied to the HASMCs at different amplitudes of 0%, 5% and 15% for a loading time of 24 h at a loading frequency of 1 Hz. When finished, the cells were collected for subsequent experiments.

### Cell transfection

Briefly, HASMCs were randomly divided into NC (HASMCs with normal culture), NC mimic (HASMCs with transfection of miR-187-3p mimic negative control), miR-187-3p mimic (HASMCs with transfection of miR-187-3p mimic), NC inhibitor (HASMCs with transfection of miR-187-3p inhibitor negative control) and miR-187-3p inhibitor (HASMCs with transfection of miR-187-3p inhibitor) groups. HASMCs were inoculated in 6-well plates 24 h prior to transfection. Then, miR-187-3p mimic, miR-187-3p inhibitor and their corresponding negative controls were transfected to HASMCs according to the instructions of Lipofectamine 2000 kit (Thermo Scientific, USA) and grouping information. After 24 h transfection, RT-qPCR was performed to detect the transfection efficiency of miR-187-3p mimic and miR-187-3p inhibitor. Thereafter, 15% CTS was applied to each group of HASMCs.

### Cell viability and apoptosis assay

The MTT kit was used to detect the proliferation of HASMCs. The treated HASMCs were seeded in a 96-well plate with 5 × 10^3^ cells per well, and cultured in an incubator at 37°C and 5% CO_2_ for 24 h. Subsequently, 20 μl MTT solution (Sigma-Aldrich) was added to each well. After 4 h incubation, 150 μl dimethyl sulfoxide was added to each well. The absorbance at 570 nm was measured using a quantitative spectrophotometer (BioTek, USA).

Detection of HASMCs apoptosis using flow cytometry. Briefly, referring to the instructions of the Annexin V-FITC/PI kit (BD Biosciences, USA), HASMCs were inoculated in 6-well plates with 3 μl Annexin-V-FITC and 5 μl propidium iodide per well, and incubated in an incubator for 15 min in the dark. Subsequently, 400 μl Annexin-FITC binding buffer was added to each well. Apoptosis was analyzed using a FACSCanto II flow cytometer (BD Biosciences).

### Transwell assay

Transwell was used to detect the migration of HASMCs. Transwells (Corning, USA) were placed in 24-well cell culture plates with a suspension of HASMCs (5 × 10^4^ cells/chamber) in the upper chamber, and 600 μl of DMEM medium containing 10% FBS in the lower chamber. The cells were incubated at 37°C for 36 h. The lower chamber HASMCs were then fixed with 4% paraformaldehyde for 30 min, and stained with 0.1% crystal violet at room temperature. After 20 min the stain was washed away and the cells were observed and photographed using a light microscope (CI60; Nikon, Japan). The number of cells was counted in 5 random fields, and then the mean was calculated.

### SmallRNA-seq

Total RNA was extracted by TRIzol kit (Qiagen, Germany) according to the manufacturer’s protocol. DNA quantification using a UV spectrophotometer (NanoDrop™ 2000; Thermo Scientific). RNA integrity was assessed using an Agilent 2100 Bioanalyzer instrument (Agilent, USA). Library construction and library inspection according to the manufacturer’s standard protocols. After passing the library check, the different libraries were pooled according to the effective concentration and the amount of data required, and then sequenced in Illumina SE50 (Illumina, USA). The thresholds for screening were absolute log_2_ Fold Change (FC)≥1 and *P* < 0.05. For readcount data of RNA-seq, please see Tab.S1.

### RT-qPCR assay

Total RNA was extracted from each group of HASMCs by referring to the TRIzol kit instructions (Qiagen). Total RNA (1 μg) was reverse transcribed into cDNA using the First-Strand cDNA Super Mix kit (TransGen Biotech, China). The obtained cDNAs were used as templates for PCR amplification to detect expression levels of miR-26a-2-3p and miR-187-3p. RT-qPCR reaction conditions: pre-denaturation at 95°C for 300 s, denaturation at 95°C for 5 s, annealing at 58°C for 20 s, extension at 72°C for 60 s, 40 cycles in total. U6 as an internal reference. The experimental results were calculated using the 2^−ΔΔCt^ method [30]. PCR primers for miR-26a-2-3p, miR-187-3p and U6 were as follows: miR-26a-2-3p: Forward primer: CCTATTCTTGATTACGAAACAA, Reverse primer: CAGTGCGTGTCGTGGAGT; miR-187-3p: Forward primer: CCATGACACAGTGTGAGACCT, Reverse primer: AACACAAGACACGAGGGGTC; U6: Forward primer: TCTAGATAATGGTGCTGATAGATGG, Reverse primer: ATAGCTGGACGCTCTGCTTC.

### Western blotting assay

Total protein was separated from each group of HASMCs using RIPA buffer, and total protein was quantified using BCA protein assay kit (Beyotime Biotechnology, CHINA). Proteins were isolated on 10% SDS-PAGE, and then was transferred to PVDF membranes. After being blocked with 5% skim milk in TBST for 1 h, membranes were incubated with primary antibody at 4°C overnight. The secondary antibodies against rabbits were then stored at room temperature for 1 h. GAPDH was used as an internal reference. Membranes were observed and photographed using a gel imager (ChemiDoc XRS+; Bio-Rad, USA) and analyzed using Image J software (version 1.0; National Institutes of Health, USA). All antibodies used in this study were purchased from Abcam, including anti-Bax (ab32503; 1:1000), anti-Bcl 2 (ab32124; 1:1000), anti-Caspase 3 (ab32351; 1:5000), anti-MMP 2 (ab92536; 1:1000), anti-MMP 9 (ab76003; 1:1000) and anti-GAPDH (ab8245; 1:4000).

### Bioinformatics analysis

Starbase [31] was used to predict the downstream target genes of miR-26a-2-3p and miR-187-3p. GO [32] and KEGG pathway [33] enrichment analysis and protein–protein interaction (PPI) network construction were performed by Metascape [34].

### Statistical analysis

All *in vitro* experiments were repeated three times, and data are expressed as mean ± SD (standard deviation). Statistical analysis was performed by SPSS 18.0 software (IBM Corporation, USA) and GraphPad Prism 8.2 (GraphPad software, USA). Statistical differences between the two groups were determined using the student *t*-test, and statistical differences between multiple groups were analyzed using a one-way analysis of variance (ANOVA). *P* < 0.05 was considered statistically significant.

## Results

### Effects of CTS on biological behavior of HASMCs

**Figure 1.**
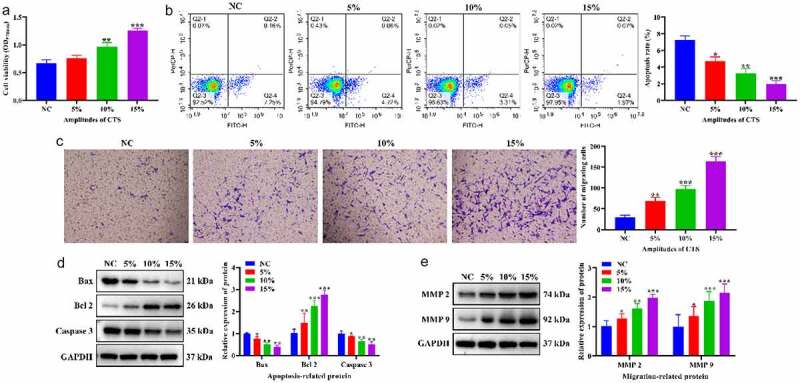
Effects of CTS on biological behavior of HASMCs. (a) MTT was used to detect HASMC proliferation. (b) Flow cytometry displayed the apoptosis level of HASMCs in each group. (c) HASMC migration was detected by Transwell. Expression levels of (d) apoptosis-related (Bax, Bcl 2 and Caspase 3) and (e) migration-related proteins (MMP 2 and MMP 9) in the cells of each group of HASMCs. In all cases, Values are mean ± SD (n = 3 for each group; *P < 0.05, **P < 0.01, ***P <0.001). CTS represents cyclic tensile strain; HASMCs stands for human aortic smooth muscle cells

To clarify the effects of CTS on proliferation, apoptosis and migration of HASMCs. HASMCs were treated with CTS at a frequency of 1 Hz and amplitudes of 10% and 15% for 24 h. MTT results confirmed that the proliferation of HASMCs was significantly increased in the 5%, 10% and 15% CTS groups compared to the NC group, and HASMC proliferation gradually increased with the enhancement of CTS amplitude. (Figure 1a). The flow cytometry results showed that, compared with the NC group, the apoptosis levels of CTS-treated HASMCs were significantly dwindled, and the higher amplitude of CTS, the lower the apoptosis levels of HASMCs (Figure 1b). Moreover, the effect of CTS on the migration of HASMCs was examined using Transwell. The results exhibited that CTS was able to increase the number of HASMCs in the lower chamber of Transwell compared to the NC group (Figure 1c). Further, the expression levels of apoptosis-associated and migration-associated proteins were detected by Western blotting. The results showed that the expression of Bax, Caspase 3, MMP2 and MMP 9 was significantly augmented and the expression of Bcl 2 was significantly diminished in CTS-treated HASMCs (Figure 1d-e). These results demonstrated that CTS facilitated the proliferation and migration of HASMCs and inhibited their apoptosis, and the effect shows the dependence of CTS amplitude.

### Differentially expressed miRNAs (DE-miRNA) in HASMCs in response to CTS

**Figure 2.**
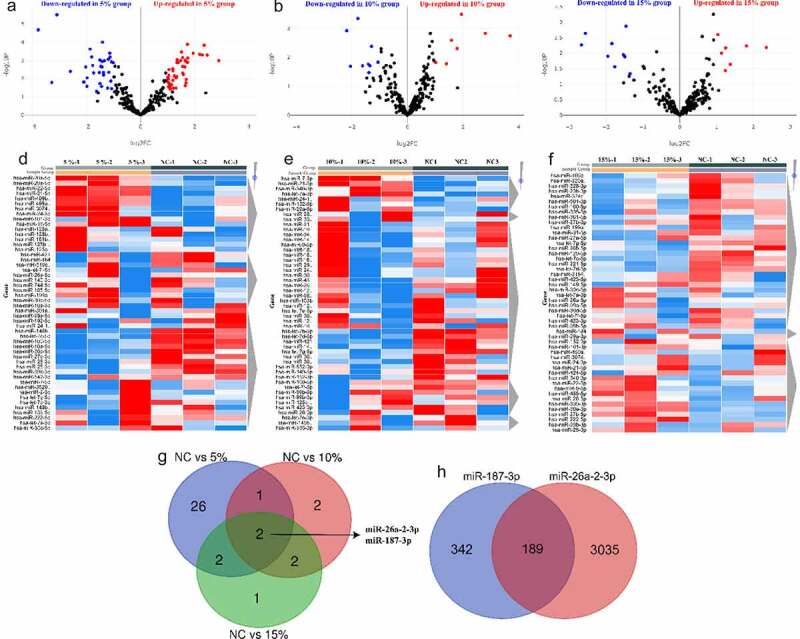
Differentially expressed miRNAs in HASMCs in response to CTS. (a-c) Volcano plots exhibited the differentially expressed miRNAs obtained in the (a) 5%, (b) 10% and (c) 15% groups compared to the NC group, respectively. The thresholds were absolute log2 FC ≥ 1 and *P* < 0.05. (d-f) Heatmap plots showed the change in expression of differentially expressed miRNAs in NC, 5%, 10% 15% groups. (g) Venn plots indicated the intersection of differentially expressed miRNAs in each group. (h) Venn plots show intersection of downstream target genes of miR-26a-2-3p and miR-187-3p

To explore the effect of CTS on miRNA expression profiles in HASMCs, we extracted mRNA from CTS-treated HASMCs for SmallRNA-seq. The screening of miRNAs was performed based on the conditions of absolute log_2_ FC≥1 and *P* < 0.05. The volcano and heatmap plots of DE-miRNAs were shown in Figure 2a-f. Compared to the NC group, there are 55 (31 up-regulated miRNAs and 24 down-regulated miRNAs), 16 (7 up-regulated miRNAs and 9 down-regulated miRNAs) and 16 (7 up-regulated miRNAs and 9 down-regulated miRNAs) DE-miRNAs in 5%, 10% and 15% CTS-treated HASMCs, respectively. Intersection results revealed that both miR-26a-2-3p and miR-187-3p were significantly highly expressed in CTS-treated HASMCs (Figure 2g). It is indicated that miR-26a-2-3p and miR-187-3p are most likely key factors in the regulation of HASMCs by CTS. Further, we predicted the target mRNA of miR-26a-2-3p and miR-187-3p by starbase database. The results verified that there were 531 downstream targets for miR-187-3p and 3224 downstream targets for miR-26a-2-3p, and there were 189 common target mRNAs (Figure 2h).

### Enrichment analysis of GO function and KEGG pathway

**Figure 3.**
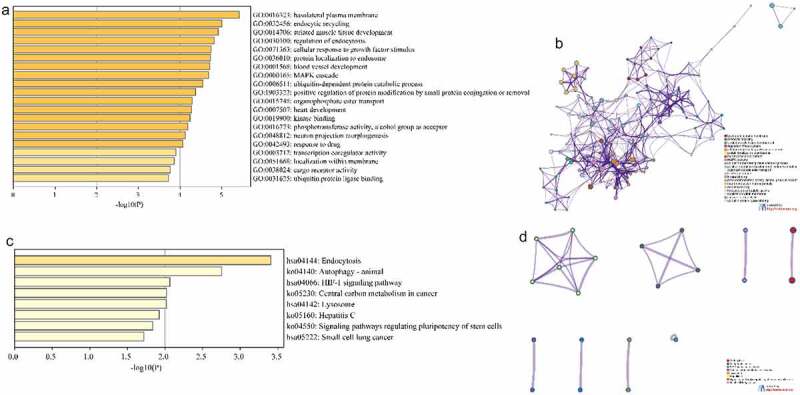
Enrichment analysis of GO function and KEGG pathway. (a and c) Bar plots showed term and pathway enriched by (a) GO and (c) KEGG, respectively. *P*-value < 0.05, a minimum count of 3, and an enrichment factor > 3.0. (b and d) Network plots exhibited the interactions between (b) each term or (d) each pathway. Each item or pathway was represented by a circle node whose size is proportional to the number of input genes in the item or pathway and whose color indicates its cluster identity

In order to determine the functions and the pathways involved of the downstream targets, we performed GO and KEGG pathway enrichment analysis Metascape. The GO enrichment results showed that a total of 251 GO terms were enriched for 189 target genes, including 183 Biological Processes, 20 Cellular Components and 48 Molecular Functions (Tab. S2). Figure 3a showed the top 20 term, mainly including ‘basolateral plasma membrane’, ‘endocytic recycling’, ‘striated muscle tissue development’, ‘regulation of endocytosis’, ‘cellular response to growth factor stimulus’ and ‘protein localization to endosome’. As shown in Figure 3b, interaction networks were constructed by correlation and similarity for the terms with high significance. KEGG enrichment showed that 189 target genes were enriched to a total of 8 KEGG pathways (Tab.S3), including ‘Endocytosis’, ‘Autophagy-animal’, ‘HIF-1 signaling pathway’, ‘Central carbon metabolism in cancer’, ‘Lysosome’, ‘Hepatitis C’, ‘Signaling pathways regulating pluripotency of stem cells’ and ‘Small cell lung cancer’ (Figure 3c). Similarly, Figure 3d showed the interaction network of the enriched KEGG pathway. These results imply that CTS can regulate cell proliferation, apoptosis and migration.

### Construction of PPI network

**Figure 4.**
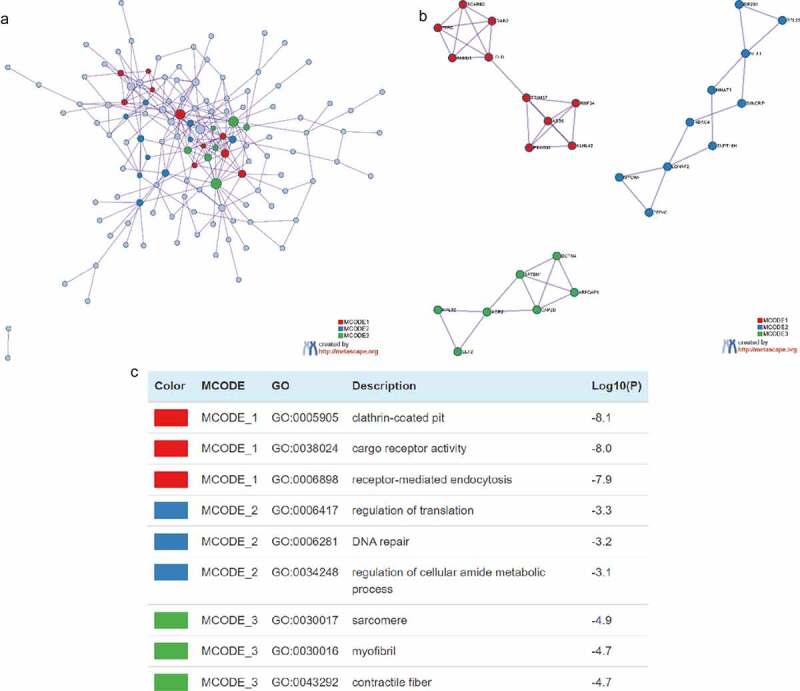
Construction of Protein-protein Interactions (PPI) network. (a) PPI network of target genes. (b) PPI network for each MCODE. (c) GO enrichment analysis was applied to each MCODE network to assign ‘meanings’ to the network components, with the top 3 best *P*-value terms retained

In order to elucidate the interactions between target genes, we further constructed PPI networks for 189 target genes by Metascape and applied the MCODE algorithm to identify protein tight junctions in the PPI networks for clustering to build functional modules (MCODE). Figure 4a displayed the fully connected interaction network of 189 target gene-associated proteins, where the three MCODEs identified from the PPI network. As shown in Figure 4b, MCODE_1 was a PPI network consisting of TFRC, SCARB2, DAB2, ARRB1, LDLR, TRIM37, ASB6, FBXO31, RNF34 and KLHL42. MCODE_2 was a PPI network composed of EIF2S1, RPL22, NFX1, MNAT1, SYNCRIP, HDAC4, SUPT16H, LONRF2, MYO9A and PPP4C. MCODE_3 was a PPI network made up of RPL32, EEF2, AGR2, CAPZB, SPTBN1, ARFGAP1 and DCTN4. Furthermore, we combined the protein interaction data with GO and KEGG pathway data to add biological significance to the MCODE identified in the protein interaction network. The results exhibited that the PPI network proteins in MCODE_1 was mainly enriched in clathrin-coated pit, cargo receptor activity and receptor-mediated endocytosis; the PPI network protein in MCODE_2 was mainly enriched in regulation of translation, DNA repair and regulation of cellular amide metabolic process; the PPI network proteins in MCODE_3 was mainly enriched in sarcomere, myofibril and contractile fibers (Figure 4c).

### Effects of CTS on biological behavior of HASMCs through miR-187-3p

**Figure 5.**
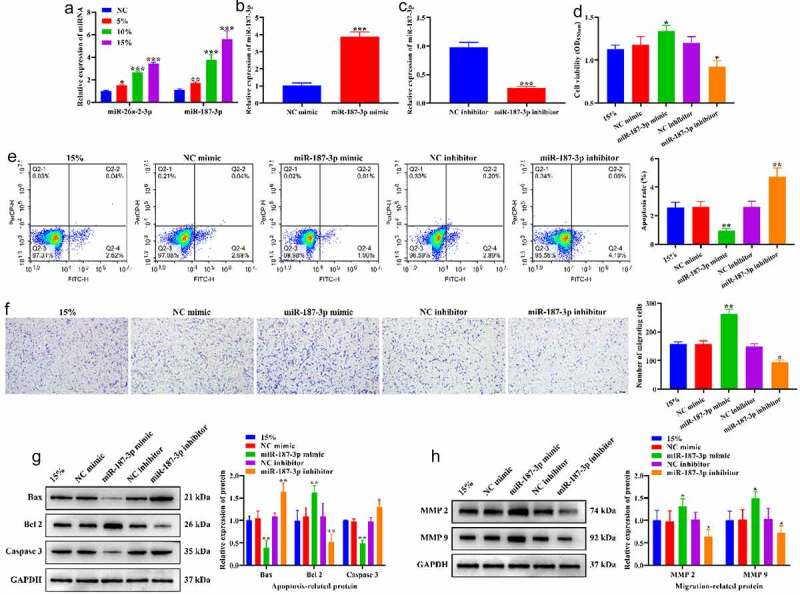
Effects of CTS on biological behavior of HASMCs through miR-187-3p. (a) Effects of CTS on the expression of miR-187-3p and miR-26a-2-3p were detected by RT-qPCR. (b-c) RT-qPCR to detect the transfection efficiency of (b) miR-187-3p mimic and (c) inhibitor. (d) MTT results demonstrate the effect of CTS on HASMC proliferation via regulating miR-187-3p. (e) The apoptosis levels of HASMCs in each group. (f) Transwell was used to detect the migration of HASMCs. Western blotting was used to detect the expression levels of (g) apoptosis related (Bax, Bcl 2 and Caspase 3) and (h) migration-related (MMP2 and MMP 9) proteins in HASMCs cells. In all cases, Values are mean ± SD (n = 3 for each group; *P < 0.05, **P < 0.01, ***P <0.001). CTS represents cyclic tensile strain; HASMCs stands for human aortic smooth muscle cells

We further validated the role of miR-26a-2-3p and miR-187-3p in the regulation of the biological behavior of HASMCs by CTS. RT-qPCR results showed that CTS significantly increased the expression levels of miR-26a-2-3p and miR-187-3p in HASMCs, and miR-187-3p was increased to a higher extent than miR-26a-2-3p (Figure 5a). Therefore, we selected miR-187-3p for subsequent validation. MiR-187-3p mimic and miR-187-3p inhibitor were applied to exogenously regulate the expression level of miR-187-3p. The results indicated that the expression of miR-187-3p was significantly increased after transfection with miR-187-3p mimic (Figure 5b) and decreased after transfection with miR-187-3p inhibitor (Figure 5c), and negative control of miR-187-3p mimic and miR-187-3p inhibitor had no effect on miR-187-3p expression in HASMCs. MTT results showed that overexpressing miR-187-3p enhanced the proliferation-promoting effect of 15% CTS on HASMCs, while knockdown of miR-187-3p generated an opposite pattern (Figure 5d). Flow cytometry results exhibited a significant reduce in HASMC apoptosis transfected with miR-187-3p mimic, and a significant augment in HASMCs apoptosis transfected with miR-187-3p inhibitor compared to 15% CTS-treated HASMCs (Figure 5e). Transwell results revealed that the number of migrating HASMCs in the miR-187-3p mimic group was significantly higher than that in the 15% group, while miR-187-3p inhibitor group was significantly lower than that in the 15% group (figure 5f). Compared to the 15% group, overexpressing miR-187-3p significantly downregulated Bax and Caspase 3 expression, and significantly upregulated Bcl2, MMP2 and MMP 9 expression, whereas knockdown of miR-187-3p prompt an opposite result (Figure 5g-h). These results suggest that CTS stimulates the proliferation and migration of HASMCs and suppresses their apoptosis by upregulating the expression of miR-187-3p.

## Discussion

Our study focused on miRNAs associated with CTS-induced phenotypic changes in VSMCs, providing some theoretical and experimental basis for exploring the mechanobiological mechanisms of stress-induced revascularization. In cardiovascular diseases such as hypertension, aortic coarctation, lumen narrowing and atherosclerosis accompanied by revascularization, these changes in vascular structure and function involve phenotypic changes in VSMCs [35,36]. Hemodynamic factors have an important influence on the morphology, structure and function of vascular cells, and the phenotype of VSMCs changes accordingly with changes in the mechanical environment, thus affecting the structure and function of the vessel wall [37,38]. In this study, we applied 5%, 10% and 15% CTS to HASMCs and detected DE-miRNAs in HASMCs in response to CTS by RNA-seq, and verified the effects of CTS and miRNAs on HASMCs phenotype. All findings demonstrate that CTS is involved in the regulation of the HASMC phenotype by upregulating the expression of miR-187-3p.

In the body, the cyclic contraction and diastole of the vessel wall due to the pulsation of the blood flow is one of the main mechanical stimuli to which the vessel is subjected. Numerous studies have now demonstrated that CTS as an external stimulus is an important factor in causing changes in the homeostasis of VSMCs [39–41]. For example, CTS was able to induce expression of thrombin on the surface of rat thoracic aortic smooth muscle cells via an integrin-related signaling pathway, and ultimately caused a phenotypic shift in smooth muscle cells [42]. CTS induces VSMCs to secrete connective tissue growth factor and promotes endothelial progenitor cell differentiation and angiogenesis [43]. CTS stimulates VSMCs alignment through redox-dependent activation of Notch3 [44]. Zhang et al. showed that high CTS-induced changes in BK and L-type calcium channels in rat thoracic aortic smooth muscle cells and led to calcium shocks [45]. This study found that CTS promoted the proliferation and migration of HASMCs and inhibited their apoptosis, which further complemented the mechanism of CTS on HASMC phenotype.

In recent years, a large number of studies have identified miRNAs as important regulators in VSMCs processes such as proliferation and angiogenesis [46,47]. For example, the exosome miR-21-3p from nicotine-treated macrophages may increase the migration and proliferation of VSMCs through its target PTEN, thereby accelerating the development of atherosclerosis [48]. Perivascular adipose tissue-derived extracellular vesicle miR-221-3p significantly enhances the proliferation and migration of VSMCs [49]. miR-93 promotes the proliferation and migration of VSMCs by targeting Mfn2 [50]. *In vivo* lentiviral delivery of miR-128-3p, a novel modulator of phenotypic switching and vascular disease in VSMCs, prevents intimal hyperplasia in a mouse model of carotid restenosis without altering important cardiovascular parameters [51]. Furthermore, Laura-Eve Mantella et al. examined the effect of CTS on HASMCs by gene chips and screened for 580 differentially expressed long non-coding RNA at the transcriptome level [52]. Similarly, we identified miRNA expression profiles in HASMCs responding to CTS by SmallRNA-seq at the transcriptome level. SmallRNA-seq results exhibited that a total of 36 DE-miRNAs were present in CTS-treated HASMCs. Notably, *in vitro* experiments indicated that CTS was able to upregulate miR-26a-2-3p and miR-187-3p expression, and miR-187-3p was increased to a higher extent than miR-26a-2-3p, which are consistent with the sequencing results. Hence, we selected miR-187-3p for subsequent validation, and found that CTS-mediated phenotypic alterations in HASMCs at least in part through regulating miR-187-3p expression. The function of miR-187-3p in cancer, reperfusion injury and sepsis has now been identified [53–56]. For example, overexpression of miR-187-3p attenuates ischemia-reperfusion-induced pain sensitivity by inhibiting the release of P2X7R and subsequently mature IL-1β in the mouse spinal cord [55] miR-187-3p inhibits metastasis and epithelial-mesenchymal transition in hepatocellular carcinoma by targeting S100A4 [57]. miR-187-3p increases gemcitabine sensitivity in breast cancer cells by downregulating FGF9 expression [58]. Unfortunately, no studies have reported the phenotypic regulation of miR-187-3p in HASMCs. In the present study, we demonstrate for the first time that miR-187-3p enhances HASMC proliferation and migration, and suppress its apoptosis, and miR-187-3p was regulated by CTS. These finding imply that miR-187-3p are strongly likely to be key regulators of CTS-mediated phenotypic changes in HASMCs, and as a potential target for CTS applications in cardiovascular disease therapy.

## Conclusion

In conclusion, this study elucidated that CTS promotes proliferation and migration and inhibits apoptosis in HASMCs, and revealed miRNAs in HASMCs that respond to CTS by RNA-seq. This suggests that miR-187-3p could be an important target of CTS leading to revascularization. These results enrich the mechanism of biomechanics on revascularization. Unfortunately, *in vivo* experiments are lacking to validate our findings in this study, and the molecular biological mechanisms by which miR-187-3p regulates HASMC phenotype remain to be further investigated.

## Supplementary Material

Supplemental Material

## Data Availability

Data is applicable after the approval of co-authhors.

## Highlights

- CTS facilitates proliferation and migration of HASMCs and reduces their apoptosis
- CTS modulates the expression of miR-187-3p
- miR-187-3p enhances proliferation and migration, and attenuates apoptosis of HASMCs
